# Lessons Learned From Cholecystectomy for a Giant Gallstone

**DOI:** 10.1002/ccr3.71651

**Published:** 2025-12-21

**Authors:** Shasha Haycock, Jordan Dewei Lee, Annie Jiao Wang, Khang Duy Ricky Le, Kristy Mansour, Bradley Bidwell

**Affiliations:** ^1^ Department of General Surgery Northeast Health Wangaratta Wangaratta Victoria Australia; ^2^ University of Melbourne Melbourne Victoria Australia

**Keywords:** cholecystectomy, cholecystitis, cholelithiasis, gallstones, laparoscopy, porcelain gallbladder

## Abstract

Giant gallstones are a rare phenomenon that may pose technical challenges for surgeons. We describe the case of an 81‐year‐old female who presented to our regional health service with acute cholecystitis and a computed tomography (CT) scan demonstrating a possible porcelain gallbladder. Her index presentation was conservatively managed with intravenous antibiotics due to body habitus and concerns for the porcelain gallbladder, with a good clinical response. She subsequently underwent an elective laparoscopic cholecystectomy which was converted to an open approach due to technical challenges. She was found to have a chronically inflamed gallbladder containing a giant gallstone. We report our successful management of a chronically inflamed gallbladder involving a giant gallstone. We also explore diagnostic considerations and surgical approaches to tackle the technical challenges we encountered in this case.

## Key Clinical Message

Giant gallstone and porcelain gallbladder may have a similar appearance on CT. Both pose technical challenges that are important to consider alongside patient comorbidities when planning patient care. While it is appropriate to attempt a laparoscopic approach, clinicians should consider early conversion to an open approach should these challenges be encountered.

## 1 Introduction

A giant gallstone, defined as a gallstone > 4 cm in diameter, is recognized as clinically significant due to heightened risk of complications and additional anticipated technical challenges when performing a laparoscopic cholecystectomy [1]. Described complications include hepatic abscess, biliary‐enteric fistula, and gallstone ileus [2, 3, 4, 5]. Predicted technical challenges include difficulty with mobilization and exposure of the critical structures of Calot's triangle secondary to adhesions and difficulty with grasping the gallbladder [6]. Notably, patients with gallbladder carcinoma have been found to have significantly larger stones, irrespective of the number of stones present, compared to both asymptomatic and symptomatic noncancer gallstone cohorts, raising the possibility of an association between giant gallstone and gallbladder carcinoma [7].

In contrast, calcification of the gallbladder wall, termed porcelain gallbladder, is an uncommon condition that affects 0.06%–0.8% of cholecystectomy specimens. In 1984, Kane et al. categorized this into three sonographic types. Type I (scleroatrophic pattern) is associated with cholelithiasis with chronic cholecystitis and carries low to no association with cancer, while type II (biconvex, hyperechoic walls with variable acoustic shadowing) and type III (irregular clumps of echoes with variable acoustic shadowing) are associated with gallbladder malignancy [8, 9]. Type I corresponds to curvilinear calcification on CT without pericholecystic mass, while type II corresponds to rim‐like calcification with pericholecystic mass. No data are available regarding the relationship of CT appearance with type III porcelain gallbladder. As type I porcelain gallbladder represents complete calcification of the gallbladder wall, it was hypothesized that there was little to no possibility of cancer (0% incidence of cancer) due to complete loss of mucosal epithelium, which is instead replaced by dense connective tissue with calcification. This contrasts with the incomplete calcification of type II and III porcelain gallbladder (12.5%–61% incidence of cancer) [9].

More recent studies, however, have failed to demonstrate a link between gallbladder wall calcification and cancer, raising questions about this traditionally accepted relationship [10]. Towfigh et al. identified 15 patients with porcelain gallbladder, all with incomplete calcification, from a total of 10,741 cholecystectomies performed between 1955 and 1998. Of these, none demonstrated evidence of cancer. Furthermore, the 88 patients who were found to have gallbladder carcinoma in this study did not demonstrate gallbladder calcification on histopathology review. Although this paper hypothesized a change in environmental factors and thus subsequent reduced carcinoma risk from the early 20th century, current recommendations and practices regarding cholecystectomy for porcelain gallbladder remain unchanged pending further research to support the contemporary suggestion that gallbladder malignancy is unrelated to porcelain gallbladders [10].

We report a case of a giant gallstone where the radiology report initially raised concern for porcelain gallbladder. Upon further review and discussion with a tertiary hepatobiliary service, this was assessed to represent instead a giant gallstone and hence deemed appropriate for regional center management.

## 2 Case History/Examination

We present the case of an independent 81‐year‐old female living alone who presented to our regional health service with a several‐day history of anorexia and non‐bilious vomiting, followed by a fall with a long lie. Of note, abdominal pain was not a reported symptom. She had a history of insulin‐dependent type 2 diabetes mellitus, hypertension, right‐sided breast cancer in remission following local excision and adjuvant chemoradiotherapy 13 years prior, with no previous abdominal surgeries.

## 3 Methods (Differential Diagnosis, Investigations and Treatment)

**FIGURE 1:**
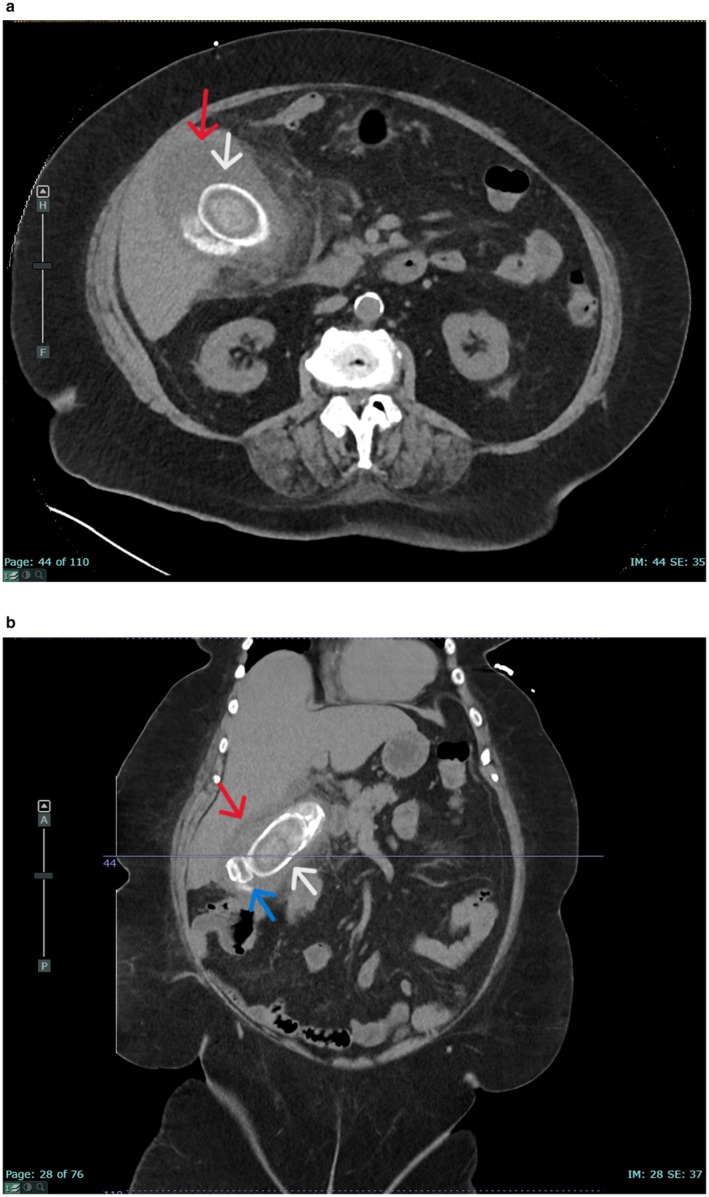
Initial computed tomography scan of the abdomen demonstrating large, calcified gallstones in (a) axial and (b) coronal views. Arrows marking calcified gallstone initially reported as porcelain gallbladder given large size and ovoid shape (white) and secondary gallstone (blue) in non‐calcified gallbladder (red).

On examination, her vital signs were within normal limits, with tenderness elicited in the right upper abdomen and an equivocal Murphy's sign. Initial biochemistry work‐up (Table 1) was significant for a moderate acute kidney injury with a creatinine of 336 μmol/L (estimated glomerular filtration rate (eGFR) of 11 mL/min) from a baseline creatinine of 78 μmol/L (eGFR 61) and elevated inflammatory markers, with a white cell count (WCC) of 26.0 × 10^9^/L, neutrophilia of 22.4 × 10^9^/L and a C‐reactive protein (CRP) of 470 mg/L. Her liver function tests revealed an elevated bilirubin of 38 μmol/L with unremarkable transaminases. Given concerns for sepsis, a computed tomography (CT) scan of the brain, chest, abdomen, and pelvis was performed, which revealed multiple gallstones within the gallbladder—the largest measuring 60 mm × 30 mm, gallbladder wall thickening, and pericholecystic fluid (Figure 1a,b).

**TABLE 1:** Biochemical markers at time of presentation compared to baseline biochemistry available from two months prior.

Laboratory test	At presentation	Baseline (2 months prior)
FBE		
Hb	143	—
WCC (Neuts)	26.0 (22.4)	—
Plts	259	—
UEC		
Na	131	141
K	3.8	4.0
Cl	91	107
Bicarb	20	27
Ur	18.7	6.7
Cr	336	61
eGFR	11	78
LFT		
Bili	38	18
ALT	34	25
AST	40	21
ALP	87	65
GGT	24	19
Albumin	25	34
CRP	470	—
Hs‐Troponin	29	—

*Note:* Abnormal values in red.

These CT findings were initially reported as suspicious for the presence of a porcelain gallbladder in addition to the findings of acute cholecystitis. Due to concerns regarding malignancy associated with a porcelain gallbladder and related technical challenges, the patient was transferred to a tertiary centre hepatobiliary unit for ongoing management. In the context of remaining clinically stable following transfer and medical comorbidities, including age and obesity, a conservative treatment approach with intravenous antibiotics was adopted acutely with a plan for elective interval cholecystectomy following medical optimization and high‐risk anesthetic review. Additionally, the diagnostic orientation changed from porcelain gallbladder to that of multiple stones including a giant gallstone within a non‐calcified gallbladder (Figure 1a,b) following further local radiological review of the CT images and discussion with a tertiary hepatobiliary unit. This shifted the therapeutic approach as, following further discussions between our team and the hepatobiliary unit regarding regional expertise and resources, it was concluded that elective cholecystectomy was appropriate at our regional centre rather than requiring specialist tertiary services. She demonstrated clinical and biochemical improvement and was subsequently discharged home with an oral course of 5 days of amoxicillin and clavulanic acid 875 mg/125 mg BD, which was completed without issue.

**FIGURE 2:**
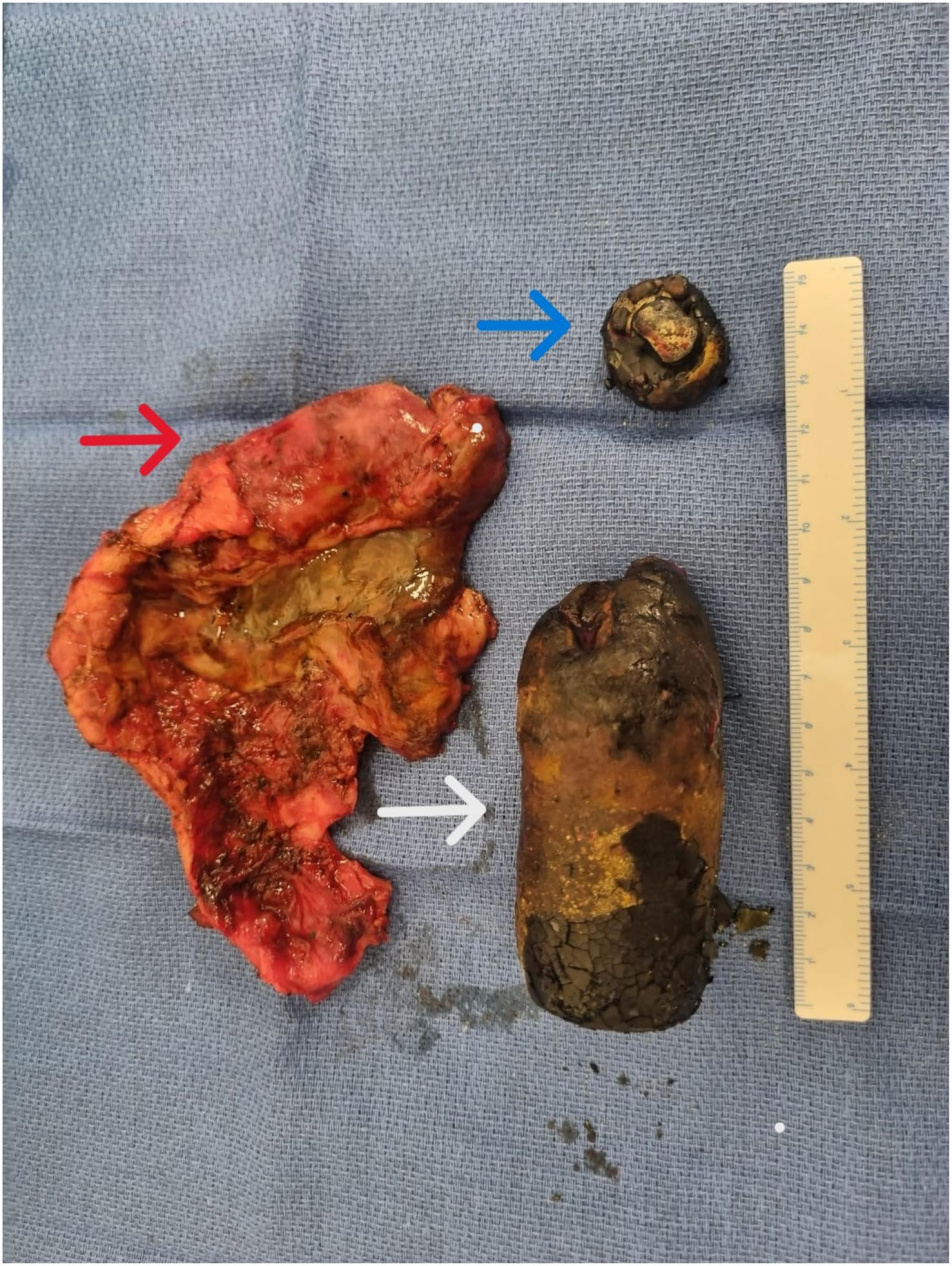
Macroscopic clinical photograph of cholecystectomy specimen (red) with removed gallstones. Arrows mark gallstones (labeled white and blue) corresponding to those visualized on initial CT as demonstrated in Figure 1.

Two months later she was admitted for elective laparoscopic cholecystectomy at our centre. Due to the preoperatively anticipated challenges, the patient was consented for a high likelihood of conversion to an open incision. This was done via an American approach with a 10 mm supraumbilical Hassan port and three 5 mm working ports, two in the right upper quadrant and one epigastric, with one primary surgeon and one assistant. At laparoscopy, significant findings included evidence of chronic cholecystitis with purulent pericholecystic fluid as well as omental and small bowel adhesions to the gallbladder and anterior abdominal wall. In addition, dissection of the hepatocystic triangle proved difficult in the context of the thick‐walled intrahepatic gallbladder and giant gallstone. This made it difficult to grip the gallbladder while significantly reducing the space required to achieve a critical view. Accordingly, an early decision was made to convert to an open procedure via a Kocher incision. The procedure was completed with the primary surgeon on the patient's right and two assistants on the patient's left, using an Omni‐Tract retractor for exposure. The adhesions were carefully dissected, with no evidence of fistulation. A plane was made between the back wall of the gallbladder, which was intentionally left behind, to allow safe extraction of the remaining gallbladder and stone. We successfully removed an enlarged, chronically inflamed gallbladder with very large gallstones (Figure 2). The total operative time was 2 h and 13 min. There were no filling defects identified on the intraoperative cholangiogram. A 14 French Blakes drain was placed and removed on day three post operation following decreased output and with no evidence of bile leak. She was continued on intravenous ceftriaxone 2 g daily and metronidazole 300 mg BD for three days following the cholecystectomy due to the purulent intraoperative pericholecystic fluid and subsequently stepped down to a five‐day course of oral amoxicillin and clavulanic acid 875 mg/125 mg BD. A culture was not obtained at the time of the operation. A right‐sided transverse abdominis plane block catheter was inserted at the time of the procedure, which was dislodged after two days. At this time her pain was well controlled, and she was transitioned to oral analgesia.

## 4 Conclusion and Results (Outcome and Follow‐Up)

Histopathology confirmed the diagnosis of chronic cholecystitis without evidence of malignancy. She underwent an unremarkable post‐operative period and was discharged on post‐operative day four. She had recovered without complication by her two‐week post‐operative follow‐up and was discharged from our service.

## 5 Discussion

Here we present the radiological findings, clinical photography of intraoperative cholecystectomy specimens, and operative learnings from a case of giant gallstone with initial concern for porcelain gallbladder. Our case reflects previous findings that porcelain gallbladder can be a difficult diagnosis and is frequently overcalled on CT, with a one in three false positive rate [8, 11]. Further review of imaging in this case demonstrated that, although ovoid in shape, the calcification present is within the gallbladder itself instead of along the gallbladder wall as expected for porcelain gallbladder. Intraoperative photos (Figure 2) demonstrate how these radio‐opaque stones correspond to operative findings.

Despite the differences of the two clinical entities, giant gallstones and porcelain gallbladders share similar surgical challenges, including limited ability to retract and mobilize the gallbladder to expose critical structures. Our operative experience in this case demonstrated the importance of early consultation with a tertiary hepatobiliary service regarding the appropriate approach and expertise needed in such cases where cholecystectomy is anticipated to be challenging and where an increased risk of gallbladder malignancy exists, warranting an oncological resection if technically feasible. In our case, we anticipated preoperatively a high chance of open operation; however, given the association of less pain, shorter hospital stay, and faster recovery with laparoscopic operation, we felt it was worthwhile attempting an initial laparoscopic approach. In this case, the predicted challenges of handling the gallbladder in addition to the body habitus of the patient resulted in the early conversion to an open operation. This highlights the importance of preoperative assessment and anticipating challenges, whereby we appropriately consented for the high likelihood of conversion to an open operation and facilitated theater set‐up accordingly. Other than consideration of completing the cholecystectomy at a tertiary center if this presentation had instead represented a case of porcelain gallbladder, our therapeutic approach would otherwise likely remain the same for the two entities: commencing with a laparoscopic approach, with recognition of the high risk of conversion to open, and preparation to complete an extended cholecystectomy if intraoperative concerns of malignancy with local invasion were associated with either condition.

The literature recommends cholecystectomy for gallstones > 3 cm even if asymptomatic, given the increased risk of complications (as mentioned above), and favors a laparoscopic approach with the possibility of conversion to open if indicated based on intraoperative findings, as in our case [1, 12]. Some surgeons, however, favor direct open cholecystectomy for the management of giant gallstones due to predicted increased inflammation severity, gallbladder wall thickening, challenges with laparoscopically grasping the gallbladder, and difficulty in the retrieval of the large gallstones, as demonstrated in our case study [13]. Despite the new debate regarding whether porcelain gallbladder represents an increased or independent risk of gallbladder cancer, the recommendation of current literature remains for early removal [14]. Consideration will need to be given for elderly and comorbid patients, weighing up perioperative risk with complications and oncological risk. Furthermore, our literature review did not identify any recommendations regarding the employment of specific techniques for oncological resection for giant gallstones or porcelain gallbladder. In this case, presentation with acute cholecystitis indicated cholecystectomy irrespective of imaging findings of giant gallstones.

## Author Contributions

**Shasha Haycock:** conceptualization, data curation, investigation, methodology, resources, writing – original draft, writing – review and editing. **Jordan Dewei Lee:** conceptualization, investigation, methodology, writing – original draft, writing – review and editing. **Annie Jiao Wang:** data curation, methodology, writing – original draft, writing – review and editing. **Khang Duy Ricky Le:** data curation, methodology, writing – original draft, writing – review and editing. **Kristy Mansour:** data curation, writing – review and editing. **Bradley Bidwell:** formal analysis, supervision, validation, writing – review and editing.

## Funding

The authors have nothing to report.

## Ethics Statement

The authors have nothing to report.

## Consent

The patient provided informed consent that was written and signed for the generation and publication of this manuscript using their de‐identified medical information.

## Conflicts of Interest

The authors declare no conflicts of interest.

## Data Availability

Data can be requested from the corresponding author when required. All relevant data have been provided in the generation of this manuscript, which is intended for open access publication.
